# Assessing the impact of HIV support groups on antiretroviral therapy adherence and viral suppression in the African cohort study

**DOI:** 10.1186/s12879-021-06390-3

**Published:** 2021-07-20

**Authors:** Prudence Mbah, Michael Iroezindu, Allahna L. Esber, Nicole Dear, Domonique Reed, Yakubu Adamu, Abdulwasiu Bolaji Tiamiyu, Samirah Sani Mohammed, Hannah Kibuuka, Jonah Maswai, John Owuoth, Emmanuel Bahemana, Julie A. Ake, Christina S. Polyak, Trevor A. Crowell, for the AFRICOS Study Group, Danielle Bartolanzo, Alexus Reynolds, Katherine Song, Mark Milazzo, Leilani Francisco, Steven Schech, Badryah Omar, Tsedal Mebrahtu, Elizabeth Lee, Kimberly Bohince, Ajay Parikh, Jaclyn Hern, Emma Duff, Kara Lombardi, Michelle Imbach, Leigh Anne Elle, Michael Semwogerere, Prossy Naluyima, Godfrey Zziwa, Allan Tindikahwa, Claire Nakazzi Bagenda, Hilda Mutebe, Cate Kafeero, Enos Baghendaghe, William Lwebuge, Freddie Ssentogo, Hellen Birungi, Josephine Tegamanyi, Paul Wangiri, Christine Nabanoba, Phiona Namulondo, Richard Tumusiime, Ezra Musingye, Christina Nanteza, Joseph Wandege, Michael Waiswa, Evelyn Najjuma, Olive Maggaga, Isaac Kato Kenoly, Barbara Mukanza, Monica Millard, Rither Langat, Aaron Ngeno, Lucy Korir, Raphael Langat, Francis Opiyo, Alex Kasembeli, Christopher Ochieng, Japhet Towett, Jane Kimetto, Brighton Omondi, Mary Leelgo, Michael Obonyo, Linner Rotich, Enock Tonui, Ella Chelangat, Joan Kapkiai, Salome Wangare, Zeddy Bett Kesi, Janet Ngeno, Edwin Langat, Kennedy Labosso, Joshua Rotich, Leonard Cheruiyot, Enock Changwony, Mike Bii, Ezekiel Chumba, Susan Ontango, Danson Gitonga, Samuel Kiprotich, Bornes Ngtech, Grace Engoke, Irene Metet, Alice Airo, Ignatius Kiptoo, Valentine Sing’oei, Winnie Rehema, Solomon Otieno, Celine Ogari, Elkanah Modi, Oscar Adimo, Charles Okwaro, Christine Lando, Margaret Onyango, Iddah Aoko, Kennedy Obambo, Joseph Meyo, George Suja, Laura Chittenden, Nnamdi Azuakola, Mfreke Asuquo, Afoke Kokogho, Samirah Sani Mohammed, Ifeanyi Okoye, Sunday Odeyemi, Aminu Suleiman, Lawrence C. Umeji, Onome Enas, Miriam Ayogu, Ijeoma Chigbu-Ukaegbu, Wilson Adai, Felicia Anayochukwu Odo, Rabi Abdu, Roseline Akiga, Helen Nwandu, Chisara Sylvestina Okolo, Ogundele Taiwo, Otene Oche Ben, Nicholas Innocent Eigege, Tony Ibrahim Musa, Juliet Chibuzor Joseph, Ndubuisi C. Okeke, Zahra Parker, Nkechinyere Elizabeth Harrison, Uzoamaka Concilia Agbaim, Olutunde Ademola Adegbite, Ugochukwu Linus Asogwa, Adewale Adelakun, Chioma Ekeocha, Victoria Idi, Rachel Eluwa, Jumoke Titilayo Nwalozie, Igiri Faith, Blessing Irekpitan Wilson, Jacinta Elemere, Nkiru Nnadi, Francis Falaju Idowu, Ndubuisi Rosemary, Amaka Natalie Uzoegwu, Theresa Owanza Obende, Ifeoma Lauretta Obilor, Doris Emekaili, Edward Akinwale, Inalegwu Ochai, Lucas Maganga, Samoel Khamadi, John Njegite, Connie Lueer, Abisai Kisinda, Jaquiline Mwamwaja, Faraja Mbwayu, Gloria David, Mtasi Mwaipopo, Reginald Gervas, Dorothy Mkondoo, Nancy Somi, Paschal Kiliba, Ephrasia Mwalongo, Gwamaka Mwaisanga, Johnisius Msigwa, Hawa Mfumbulwa, Peter Edwin, Willyhelmina Olomi

**Affiliations:** 1 U.S. Military HIV Research Program, Walter Reed Army Institute of Research, Silver Spring, MD USA; 2 HJF Medical Research International, Abuja, Nigeria; 3 Henry M. Jackson Foundation for the Advancement of Military Medicine Inc., Bethesda, MD USA; 4 U.S. Army Medical Research Directorate – Africa, Abuja, Nigeria; 5 Makerere University Walter Reed Project, Kampala, Uganda; 6 HJF Medical Research International, Kericho, Kenya; 7 U.S. Army Medical Research Directorate – Africa, Kisumu, Kenya; 8 HJF Medical Research International, Kisumu, Kenya; 9 HJF Medical Research International, Mbeya, Tanzania

**Keywords:** Africa, ART adherence, HIV, Support group, Viral suppression

## Abstract

**Background:**

Support groups for people living with HIV (PLWH) may improve HIV care adherence and outcomes. We assessed the impact of support group attendance on antiretroviral therapy (ART) adherence and viral suppression in four African countries.

**Methods:**

The ongoing African Cohort Study (AFRICOS) enrolls participants at 12 clinics in Kenya, Uganda, Tanzania, and Nigeria. Self-reported attendance of any support group meetings, self-reported ART adherence, and HIV RNA are assessed every 6 months. Logistic regression models with generalized estimating equations were used to estimate adjusted odds ratios (aORs) and 95% confidence intervals (95% CIs) for support group attendance and other factors potentially associated with ART adherence and viral suppression.

**Results:**

From January 2013 to December 1, 2019, 1959 ART-experienced PLWH were enrolled and 320 (16.3%) reported any support group attendance prior to enrollment. Complete ART adherence, with no missed doses in the last 30 days, was reported by 87.8% while 92.4% had viral suppression <1000copies/mL across all available visits. There was no association between support group attendance and ART adherence in unadjusted (OR 1.01, 95% CI 0.99–1.03) or adjusted analyses (aOR 1.00, 95% CI 0.98–1.02). Compared to PLWH who did not report support group attendance, those who did had similar odds of viral suppression in unadjusted (OR 0.99, 95% CI 0.978–1.01) and adjusted analyses (aOR 0.99, 95% CI 0.97–1.01).

**Conclusion:**

Support group attendance was not associated with significantly improved ART adherence or viral suppression, although low support group uptake may have limited our ability to detect a statistically significant impact.

## Background

Sub-Saharan Africa (SSA) carries a disproportionate burden of human immunodeficiency virus (HIV) infections, accounting for two-thirds of the global burden of the disease [1]. Improved access to antiretroviral therapy (ART) over the last decade has significantly improved treatment outcomes and the quality of life of people living with HIV (PLWH) in SSA [2]. However, substantial gaps in HIV testing and ART access impeded the achievement of the previously set Joint United Nations Program on HIV and AIDS (UNAIDS) ‘90–90-90 targets’ to diagnose 90% of all PLWH, provide ART for 90% of those diagnosed and achieve viral suppression for 90% of those treated, by 2020 [3]. Effective strategies that promote healthcare engagement and ART adherence are needed to achieve the currently set ‘95–95-95 targets’ by 2030 [4].

Psychosocial support in the form of support groups has been recognized by the World Health Organization (WHO) as a useful strategy for optimizing HIV care [5]. A support group for PLWH is a group of people who come together to share challenges and experiences of living with the virus, without being judged, blamed, stigmatized or isolated [6]. Despite the existence of basic guidelines that underpin the formation and operation of HIV support groups, there appears to be no standardization in the services they provide and they may vary in the level of formality, location of meetings, number of participants, frequency of attendance, and topics discussed [7, 8]. The broad topics typically addressed by HIV support groups include disclosure, mitigating stigma, living positively with HIV, and building relationships [6–8]. Living positively with HIV incorporates acceptance of the diagnosis, handling health challenges, nutritional support, ART adherence and overcoming psychological challenges. So far, the literature is replete with studies that have evaluated the impact of support group participation on psychosocial aspects of HIV care [9–13]. In these prior studies, HIV support group attendance was found to be an effective intervention for reducing stigma, discrimination, depression, and for achieving higher levels of active coping, self-esteem, better adjustment to one’s HIV status as well as better management of partner’s reaction to disclosure [9, 11, 12]. However, there were mixed findings regarding rate of partner disclosure among PLWH who attended support groups compared to those who did not [10–12]. In the context of scaling up of ART in SSA and the ‘95–95-95 targets’, looking beyond potential psychosocial benefits by assessing the impact of HIV support groups on ART adherence and treatment outcomes is crucial.

So far, there is limited literature on the impact of HIV support groups on ART adherence and treatment outcomes. In a systematic review that generally evaluated the impact of HIV support groups on clinical outcomes, support group participation was reported to be consistently beneficial on a range of morbidity outcomes including reduced frequency of HIV-related somatic and psychological symptoms, improved access to ART, ART adherence and treatment success, and moderately impactful on improving mortality and quality of life [14]. Treatment success was measured as time to treatment failure or reduced risk of detectable viremia or change in CD4 count. An association between support group participation and improved ART adherence and viral suppression was specifically demonstrated by observational studies from SSA included in the systematic review [15–17]. In a study in Kenya that followed PLWH for 1 year after initiation of ART, participation in three or more support groups was associated with better ART adherence and a significant reduction in the risk of viral failure [16]. In South Africa, support group participants were significantly more likely to have an undetectable viral load at 12 months than those who did not participate in a support group and serial support group participation improved the likelihood of viral suppression after 24 months of ART [15]. Conversely, peer support did not show any impact on viral outcomes after 2 years of follow-up in Vietnam [18]. Although findings from the systematic review consistently reported morbidity benefits, the reviewers rated the overall quality of evidence as fair based on the methodological limitations of many of the studies such as small sample sizes, cross-sectional analyses, qualitative design, and confounding variables, which led to their recommendation of additional research to understand the benefits of support group participation on HIV care and treatment.

Understanding the impact of support group participation on ART adherence and viral suppression in a large multi-country prospective cohort of PLWH in Africa could inform strategies for improving HIV treatment outcomes. We described HIV support group attendance at enrollment into the African Cohort Study (AFRICOS) and evaluated the impact of support group attendance on ART adherence and viral suppression across different time points.

## Methods

### Study population and procedures

AFRICOS, established in 2013, is an ongoing longitudinal observational study that enrolls PLWH and a smaller group of adults without HIV aged 18 years or older at 12 PEPFAR-supported clinical care sites in Uganda, Kenya, Tanzania, and Nigeria as previously described [19]. Most PLWH were invited to the study based on random selection from existing clinic patient lists (stratified by gender and ART status) or new enrollees to the clinic, while a minority (less than 5%) are recruited from other HIV studies performed by our group locally to facilitate long-term follow-up. Individuals were eligible if they were aged ≥18 years and consented to data and specimen collection. An additional inclusion criterion for HIV-infected individuals was the ongoing receipt of HIV care at the enrolling clinic. We excluded individuals who were pregnant at enrollment. At enrollment and every 6 months thereafter, participants undergo medical history-taking, physical examination, and laboratory assessments. For PLWH, this includes HIV RNA quantification using any of several different polymerase chain reaction testing platforms as previously described [20]. Participants also complete a broad demographic and socio-behavioral questionnaire. All participants provided written informed consent. Relevant institutional review boards provided approval as highlighted under the declaration section.

### Data collection and measures

Demographic variables including clinical care site, age, sex, education status, employment status, and marital status were collected by self-report at enrollment. History of alcohol use (dichotomized as “yes” or “no”) was also obtained at enrollment. WHO clinical stage at the time of HIV diagnosis, time on ART, and ART regimen were extracted from the medical records. The study database was used to identify participants who did and did not report support group attendance at each study visit. Both participants who reported support group attendance prior to enrollment and those who did not were followed longitudinally. Participants who joined a support group after the enrollment visit were categorzied as such for subsequent observation times. Due to variable time since study initiation across participating sites, the maximum numbers of possible completed visits were 14 in Uganda, 13 in South Rift Valley (SRV), Kenya, and 12 at all other sites.

Participants were asked “how often have you attended an HIV support group?” Self-reported support group attendance was classified as “yes” if they indicated any of the following frequencies of support group attendance: “less than once a month”, “once a month”, “more than once a month”, or “several times within a month”. It was classified as “no” if the response was “not at all”. ART adherence was based on self-report during study visits dichotomized as “complete” (not missing a dose in the last 30 days) or “incomplete” (missing one or more doses in the last 30 days). Participants were asked “Do you have a treatment supporter or treatment companion, that is, someone who supports you in taking your ARVs?”, and the response was captured as yes vs no. Viral suppression was defined as HIV RNA < 1000 copies/mL in participants on ART. PLWH with HIV RNA ≥1000 copies/mL and on ART for ≥6 months were classified as having viral failure.

### Overview of support group activities at AFRICOS sites

At all the sites, ART clinic staff create awareness about the existence of HIV support groups during daily health talks, prior to ART initiation, and/or during adherence counselling. PLWH are usually referred to support groups by ART clinic staff when a need is identified. The existence of a general support group is common to all sites. To a variable extent, most sites also have a number of need-specific support groups for discordant partners, adolescents, pregnant women, men, and PLWH with treatment failure. Support group meetings are more or less facility-based across the sites. However, community-based meetings are also held in Kisumu West, Kenya while the Uganda site occasionally embarks on one-on-one support group field visits. In Tanzania, some community-based meetings occur but are not under the direct supervision of the clinic. All attendees are required to register as members. Periodicity of meetings is variable but on average tends to be monthly. In Kisumu West, Kenya the meetings are alternated bi-weekly between the facility and the community. A snack is usually served during the meetings.

The attendees are coordinated by members elected or appointed to play leadership roles while healthcare workers, clinic managers or social workers oversee activities and provide guidance as needed. During support group meetings, general health education on HIV care and treatment is provided. Topics discussed may include status disclosure, ART adherence, healthy nutrition, exercise, handling challenges, appropriate and consistent use of condoms, among others. They also share life experiences about positive living with HIV. In addition, the groups provide active services such as adherence counselling, psychosocial support towards status acceptance/status disclosure, managing reported stigmatization or discrimination, financial support, skill acquisition trainings, and matchmaking for single or widowed PLWH. In some sites such as Kayunga, Uganda, they may undertake home visits for PLWH who missed clinic visits including those deemed lost to follow-up.

### Statistical analyses

Participant characteristics at study enrollment were compared between those who reported support group attendance and those who did not using Chi-square or Fisher’s exact test as appropriate. Chi-square tests were used to assess significant differences in support group attendance across and within sites over time. Logistic regression with generalized estimating equations was used to estimate unadjusted and adjusted odds ratios (ORs) and 95% confidence intervals (CIs) for associations between support group attendance with ART adherence and viral suppression. Sensitivity analyses were performed examining the association of support group attendance (yes vs. no) at the current and previous visit with viral suppression and ART adherence across all visits except for enrollment. A *p*-value < 0.05 was considered statistically significant. All analyses were performed using SAS 9.3 (SAS, Cary, NC) and Stata 15.0 (StataCorp, College Station, TX).

## Results

### Characteristics of the study population

From January 2013 to December 1, 2019, a total of 2925 PLWH enrolled into AFRICOS of which 2025 (69.2%) were on ART. Of these, 1959 had complete data for all variables at enrollment. Analyses were restricted to PLWH on ART with complete data unless otherwise stated. If a participant was not on ART at enrollment, but was commeced on ART at a later visit, that visit was included. The median number of study visits in the entire cohort was 5 (interquartile range [IQR] 3–8). Most participants were female (56.3%), married (57.3%), and currently unemployed (64.6%). Participants had a median age of 40.1(IQR 32.9–47.9) years. WHO clinical stage I (34.5%) was the most common at the time of HIV diagnosis, closely followed by stages II (30.6%) and III (27.4%). The median duration on ART was 3.1 (IQR 0.7–5.9) years and 20.4% of participants were on ART for < 6 months. A combination of tenofovir/lamivudine/efavirenz [TLE] (47.5%) was the most common ART regimen followed by zidovudine/nevirapine/lamivudine [AZT/NVP/3TC] (25.2%), while 6.9% of participants were on a PI-based regimen. The majority of participants (65.4%) reported having an ART treatment supporter (Table 1).

**Table 1:** Characteristics of people living with HIV on ART at enrollment by HIV support group attendance

Characteristics	All participants (*n* = 1959)	Did not attend support group (*n* = 1639)	Attended support group (*n* = 320)	*p-value
**Study site**				***< 0.001***
Kayunga, Uganda	218 (11.1%)	176 (10.7%)	42 (13.1%)	
South Rift Valley, Kenya	782 (39.9%)	625 (38.1%)	157 (49.1%)	
Kisumu West, Kenya	391 (20.0%)	354 (21.6%)	37 (11.6%)	
Mbeya, Tanzania	370 (18.9%)	323 (19.7%)	47 (14.7%)	
Abuja & Lagos Nigeria	198 (10.1%)	161 (9.8%)	37 (11.6%)	
**Age** (years)				***< 0.01***
18–29	334 (17.0%)	289 (17.6%)	45 (14.1%)	
30–39	637 (32.5%)	550 (33.6%)	87 (27.2%)	
40–49	602 (30.7%)	496 (30.3%)	106 (33.1%)	
50+	386 (19.7%)	304 (18.5%)	82 (25.6%)	
**Sex**				*0.80*
Male	857 (43.7%)	715 (43.6%)	142 (44.4%)	
Female	1102 (56.3%)	924 (56.4%)	178 (55.6%)	
**Education**				*0.43*
None or some primary	625 (31.9%)	524 (32.0%)	101 (31.6%)	
Primary or some secondary	759 (38.7%)	643 (39.2%)	116 (36.3%)	
Secondary and above	575 (29.4%)	472 (28.8%)	103 (32.2%)	
**Currently employed**				***< 0.01***
No	1265 (64.6%)	1081 (66.0%)	184 (57.5%)	
Yes	694 (35.4%)	558 (34.0%)	136 (42.5%)	
**Marital status**				*0.20*
Not married	837 (42.7%)	690 (42.1%)	147 (45.9%)	
Married	1122 (57.3%)	949 (57.9%)	173 (54.1%)	
**Consume alcohol**				*0.12*
No	1638 (83.6%)	1361 (83.0%)	277 (86.6%)	
Yes	321 (16.4%)	278 (17.0%)	43 (13.4%)	
**ART treatment supporter**				*0.22*
No	677 (34.6%)	576 (35.1%)	101 (31.6%)	
Yes	1282 (65.4%)	1063 (64.9%)	219 (68.4%)	
**WHO stage at time of HIV diagnosis**				*0.20*
I	676 (34.5%)	582 (35.5%)	94 (29.4%)	
II	599 (30.6%)	486 (29.7%)	113 (35.3%)	
III	537 (27.4%)	448 (27.3%)	89 (27.8%)	
IV	98 (5.0%)	83 (5.1%)	15 (4.7%)	
Unknown	49 (2.5%)	40 (2.4%)	9 (2.8%)	
**Duration on ART**				***< 0.001***
< 6 months	399 (20.4%)	379 (23.1%)	20 (6.3%)	
6 months to < 2 years	406 (20.7%)	361 (22.0%)	45 (14.1%)	
2 to < 4 years	336 (17.2%)	284 (17.3%)	52 (16.3%)	
4+ years	818 (41.8%)	615 (37.5%)	203 (63.4%)	
**ART regimen**				***< 0.001***
AZT/NVP/3TC	493 (25.2%)	378 (23.1%)	115 (35.9%)	
AZT/EFV/3TC	167 (8.5%)	141 (8.6%)	26 (8.1%)	
TDF/NVP/3TC	173 (8.8%)	139 (8.5%)	34 (10.6%)	
PI-based	135 (6.9%)	97 (5.9%)	38 (11.9%)	
TLE	930 (47.5%)	830 (50.6%)	100 (31.3%)	
Other	61 (3.1%)	54 (3.3%)	7 (2.2%)	

From January 2013 to December 1, 2019, a total of 1959 PLWH on ART enrolled into AFRICOS and 320 (16.3%) reported any support group attendance prior to enrollment. Those who reported support group attendance differed significantly from those who did not by site, age, employment status, duration on ART, and ART regimen

*Statistically significant *p*-values in bold

As of the most recent participants’ follow-up visit, there were 2662 PLWH on ART. The median age was 42.4 (IQR 35.2–50.2) and 58.0% were female. The median duration on ART was 4.9 (IQR 3.1–8.7) and 2.8% were on ART for < 6 months. PLWH on TLE (39.3%) and other regimen (37.9%) were predominant, while 11.9% were on a PI-based regimen. Having a treatment supporter was documented in 60.6% of participants.

### HIV support group attendance

**Fig. 1:**
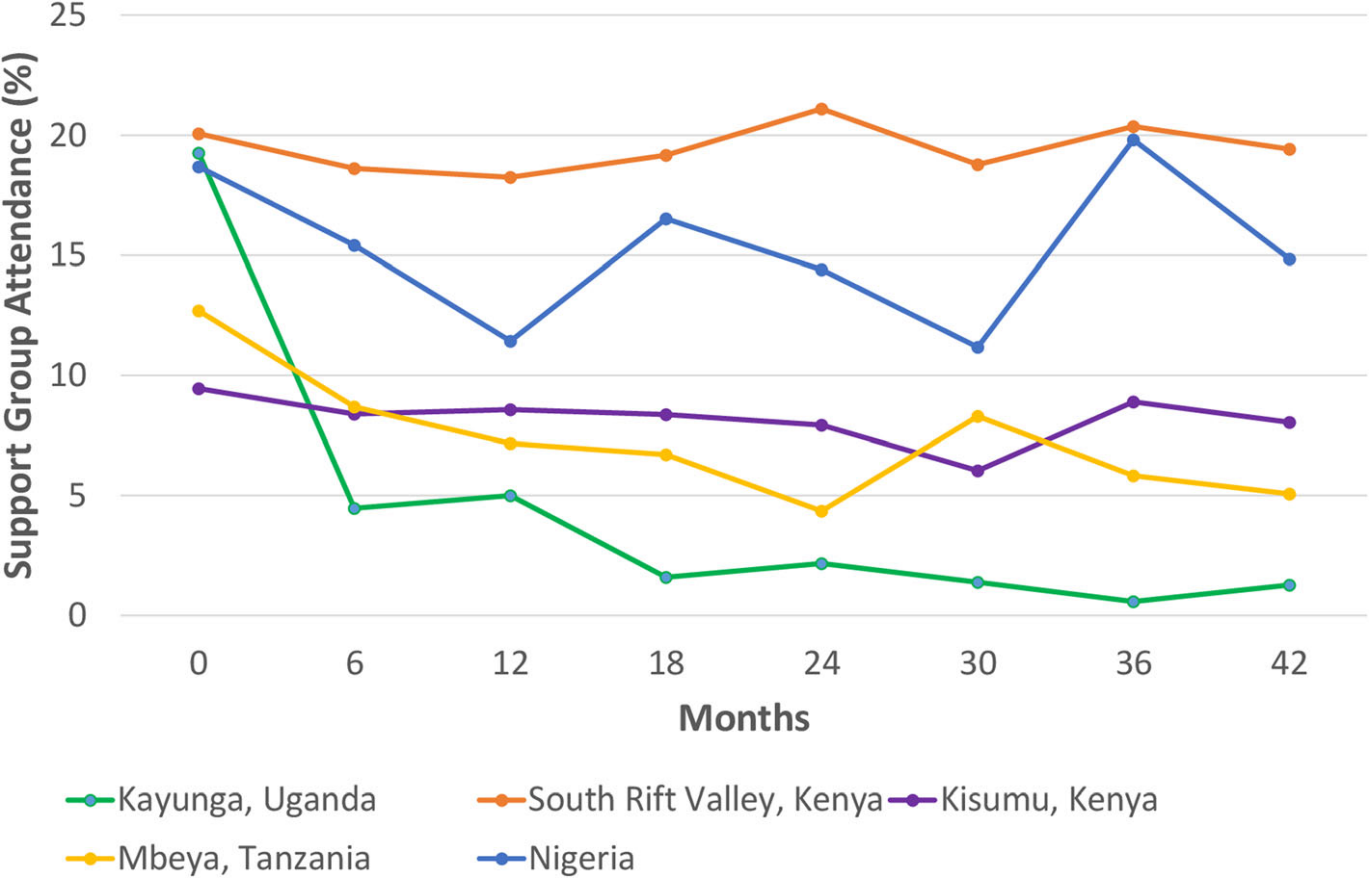
HIV support group attendance in the African Cohort Study across different time points, from enrollment visit (month 0*) to month 42 (visit 8). Due to progressively small sample sizes in later visits, the data was censored at month 42 (visit 8) follow-up with 156 participants reporting support group attendance at this time point in the entire cohort. Chi-square tests were used to assess significant differences in self-reported support group attendance across and within sites over time. There was a statistically significant difference in inter-site self-reported support group attendance across different time points, *p* < 0.01. Significant within-site difference in self-reported support group attendance was observed in Kayunga, Uganda (*p* < 0.01), SRV, Kenya (*p* < 0.01), Tanzania (*p* = 0.01), and Nigeria (*p* = 0.02) but not in Kisumu West, Kenya (*p* = 0.63). *Month zero (0) is not the same time point for all participants, as it depended on their enrollment date (ranging from Jan 2013 to Nov 2019)

Of the 1959 PLWH included, 320 (16.3%) reported any support group attendance prior to enrollment. At the most recent participants’ follow-up visit, support group attendance was reported by 180/2662 (6.8%). Site-specific support group attendance across different time points ranging from enrollment visit (month 0) to month 42 follow-up is shown in Fig. 1. There was a statistically significant difference in inter-site support group attendance across different time points (*p* < 0.01). Within-site analyses showed a statistically significant difference in support group attendance across different time points in Kayunga, Uganda (*p* < 0.01), South Rift Valley, Kenya (*p* < 0.01), Tanzania (*p* = 0.01) and Nigeria (*p* = 0.02) but not in Kisumu West, Kenya (*p* = 0.63) (Fig. 1).

### ART adherence

Complete ART adherence was reported by 87.8% of participants across all available visits. There was no association between support group attendance and ART adherence in unadjusted (OR 1.01, 95% CI 0.99–1.03) and adjusted analyses (aOR 1.00, 95% CI 0.98–1.02) (Table 2).

**Table 2:** Unadjusted and adjusted analyses of factors associated with viral suppression and ART adherence among people living with HIV on ART

	Viral suppression		ART adherence	
	Unadjusted OR (95% CI)	Adjusted OR (95% CI)	Unadjusted OR (95% CI)	Adjusted OR (95% CI)
**Support group attendance**				
No	Ref	–	Ref	–
Yes	0.99 (0.97–1.01)	0.99 (0.97–1.01)	1.01 (0.99–1.03)	1.00 (0.98–1.02)
**Study site**				
Kayunga, Uganda	Ref	–	Ref	–
South Rift Valley, Kenya	**0.96 (0.95–0.98)**	**0.96 (0.94–0.99)**	**1.17 (1.13–1.20)**	**1.14 (1.10–1.18)**
Kisumu West, Kenya	**0.97 (0.95–0.99)**	0.99 (0.96–1.01)	**1.10 (1.06–1.14)**	**1.09 (1.09–1.05)**
Mbeya, Tanzania	**0.88 (0.86–0.91)**	**0.90 (0.87–0.93)**	**1.07 (1.03–1.11)**	**1.06 (1.02–1.11)**
Abuja & Lagos Nigeria	**0.91 (0.87–0.94)**	**0.90 (0.86–0.94)**	**0.88 (0.83–0.94)**	**0.88 (0.82–0.94)**
**Age** (years)				
18–29	Ref	–	Ref	–
30–39	**1.03 (1.00–1.06)**	1.02 (1.00–1.05)	**1.03 (1.00–1.07)**	**1.03 (1.00–1.06)**
40–49	**1.07 (1.04–1.09)**	**1.06 (1.03–1.08)**	**1.07 (1.04–1.11)**	**1.06 (1.03–1.10)**
50+	**1.07 (1.04–1.10)**	**1.06 (1.03–1.09)**	**1.08 (1.05–1.12)**	**1.06 (1.02–1.09)**
**Sex**				
Male	Ref	–	Ref	–
Female	1.02 (1.00–1.03)	**1.03 (1.01–1.05)**	1.01 (0.99–1.03)	1.01 (0.99–1.03)
**Education**				
None or some primary	Ref	–	Ref	–
Primary or some secondary	**0.98 (0.96–0.99)**	1.00 (0.99–1.02)	1.02 (0.99–1.04)	1.01 (1.00–1.03)
Secondary and above	**0.96 (0.94–0.98)**	0.99 (0.97–1.02)	0.99 (0.97–1.02)	1.01 (0.99–1.03)
**Currently employed**				
No	Ref	–	Ref	–
Yes	**1.02 (1.01–1.04)**	1.01 (1.00–1.03)	**0.94 (0.93–0.96)**	1.00 (0.98–1.01)
**Marital status**				
Not married	Ref	–	Ref	–
Married	**1.02 (1.00–1.03)**	1.01 (1.00–1.03)	1.01 (0.99–1.03)	1.00 (0.99–1.02)
**Consume alcohol**				
No	Ref	–	Ref	–
Yes	**0.98 (0.96–1.00)**	1.00 (0.98–1.02)	**0.88 (0.85–0.91)**	**0.89 (0.86–0.92)**
**ART treatment supporter**				
No	Ref	–	Ref	–
Yes	1.01 (1.00–1.02)	1.01 (0.99–1.01)	**1.01 (1.00–1.03)**	1.01 (.099–1.02)
**WHO stage at time of HIV diagnosis**				
I	Ref	–	Ref	–
II	1.00 (0.98–1.02)	0.99 (0.97–1.01)	0.99 (0.97–1.02)	1.00 (0.98–1.03)
III	**0.97 (0.95–0.99)**	**0.96 (0.94–0.99)**	**1.03 (1.01–1.06)**	1.02 (1.00–1.04)
IV	0.99 (0.95–1.03)	0.98 (0.93–1.03)	**1.08 (1.04–1.12)**	**1.06 (1.02–1.10)**
Unknown	0.97 (0.90–1.04)	0.96 (0.88–1.05)	**1.06 (1.01–1.12)**	1.04 (0.99–1.09)
**Duration on ART**				
< 6 months	Ref	–	Ref	–
6 months to < 2 years	**1.09 (1.06–1.12)**	**1.08 (1.05–1.11)**	0.98 (0.96–1.01)	0.98 (0.95–1.00)
2 to < 4 years	**1.10 (1.07–1.14)**	**1.08 (1.05–1.11)**	1.01 (0.98–1.04)	0.99 (0.96–1.02)
4+ years	**1.11 (1.07–1.14)**	**1.07 (1.04–1.10)**	**1.04 (1.01–1.07)**	1.02 (0.99–1.05)
**ART regimen**				
AZT/NVP/3TC	**0.96 (0.94–0.98)**	**0.96 (0.94–0.98)**	**0.97 (0.95–0.99)**	0.98 (0.95–1.00)
AZT/EFV/3TC	1.01 (0.98–1.04)	1.01 (0.99–1.04)	0.98 (0.94–1.01)	0.97 (0.94–1.01)
TDF/NVP/3TC	**0.96 (0.93–0.99)**	**0.95 (0.92–0.98)**	1.00 (0.97–1.03)	1.00 (0.97–1.03)
PI-based	**1.29 (1.24–1.34)**	**1.27 (1.22–1.32)**	**1.04 (1.00–1.07)**	1.03 (1.00–1.06)
TLE	0.99 (0.97–1.00)	1.00 (0.98–1.01)	0.99 (0.97–1.01)	1.01 (0.99–1.03)
Other	Ref	–	Ref	–

Logistic regression models with generalized estimating equations were used to estimate unadjusted and adjusted odds ratios and 95% confidence intervals for the impact of self-reported support group attendance on ART adherence and viral suppression. Statistically significant ORs and 95% CI are in bold

*OR* odds ratio, *CI* confidence interval

Sensitivity analyses showed that compared to PLWH who did not report support group attendance at the current and previous visit, those who did were significantly more likely to report complete ART adherence across all visits in the unadjusted model (OR 1.02, 95% CI 1.00–1.05), but this association was not observed after controlling for potential confounders (aOR 1.01, 95% CI 1.00–1.03) (Table 3).

**Table 3:** Sensitivity analyses examining support group attendance reported at the current and previous visit with viral suppression and ART adherence across all visits except for enrollment

	Viral suppression		ART adherence	
	Unadjusted OR (95% CI)	Adjusted OR (95% CI)	Unadjusted OR (95% CI)	Adjusted OR (95% CI)
**Support group attendance**				
No	Ref	–	Ref	–
Yes	0.99 (0.97–1.01)	0.99 (0.97–1.01)	**1.02 (1.00–1.05)**	1.01 (1.00–1.03)
**Study site**				
Kayunga, Uganda	Ref	–	Ref	–
South Rift Valley, Kenya	**0.96 (0.95–0.98)**	**0.97 (0.94–0.99)**	**1.18 (1.14–1.21)**	**1.14 (1.10–1.18)**
Kisumu West, Kenya	**0.97 (0.95–0.99)**	0.98 (0.95–1.01)	**1.11 (1.07–1.15)**	**1.09 (1.05–1.13)**
Mbeya, Tanzania	**0.90 (0.88–0.92)**	**0.91 (0.88–0.94)**	**1.06 (1.02–1.10)**	**1.05 (1.01–1.10)**
Abuja & Lagos Nigeria	**0.92 (0.88–0.96)**	**0.92 (0.87–0.96)**	**0.89 (0.83–0.95)**	**0.88 (0.82–0.94)**
**Age** (years)				
18–29	Ref	–	Ref	–
30–39	**1.03 (1.00–1.05)**	1.02 (1.00–1.05)	**1.04 (1.01–1.08)**	1.03 (1.00–1.07)
40–49	**1.06 (1.03–1.08)**	**1.06 (1.03–1.08)**	**1.08 (1.05–1.12)**	**1.06 (1.03–1.10)**
50+	**1.06 (1.03–1.09)**	**1.06 (1.03–1.10)**	**1.09 (1.05–1.13)**	**1.06 (1.02–1.10)**
**Sex**				
Male	Ref	–	Ref	–
Female	1.02 (1.00–1.03)	**1.03 (1.01–1.05)**	1.01 (0.98–1.03)	1.01 (0.99–1.03)
**Education**				
None or some primary	Ref	–	Ref	–
Primary or some secondary	**0.98 (0.96–0.99)**	1.00 (0.98–1.01)	1.01 (1.00–1.03)	1.01 (0.99–1.03)
Secondary and above	**0.96 (0.94–0.98)**	0.99 (0.97–1.01)	1.00 (0.97–1.02)	1.01 (0.99–1.03)
**Currently employed**				
No	Ref	–	Ref	–
Yes	**1.02 (1.01–1.03)**	1.01 (0.99–1.03)	**0.94 (0.93–0.96)**	1.00 (0.98–1.02)
**Marital status**				
Not married	Ref	–	Ref	–
Married	1.01 (1.00–1.03)	1.01 (1.00–1.03)	1.01 (1.00–1.03)	1.01 (0.99–1.03)
**Consume alcohol**				
No	Ref	–	Ref	–
Yes	0.98 (0.96–1.00)	1.00 (0.98–1.02)	**0.89 (0.86–0.92)**	**0.90 (0.87–0.93)**
**ART treatment supporter**				
No	Ref	–	Ref	–
Yes	1.01 (1.00–1.02)	1.00 (0.99–1.01)	1.01 (1.00–1.03)	1.00 (0.99–1.02)
**WHO stage at time of HIV diagnosis**				
I	Ref	–	Ref	–
II	0.99 (0.98–1.01)	0.99 (0.97–1.01)	0.99 (0.96–1.01)	1.00 (0.98–1.03)
III	**0.97 (0.95–0.99)**	**0.97 (0.94–0.99)**	**1.03 (1.01–1.06)**	1.02 (1.00–1.05)
IV	0.99 (0.95–1.03)	0.98 (0.94–1.03)	**1.08 (1.04–1.12)**	**1.07 (1.03–1.10)**
Unknown	0.96 (0.89–1.04)	0.96 (0.88–1.04)	**1.07 (1.01–1.12)**	1.04 (0.99–1.09)
**Duration on ART**				
< 6 months	Ref	–	Ref	–
6 months to < 2 years	0.99 (0.97–1.01)	0.99 (0.97–1.01)	1.02 (0.97–1.06)	1.01 (0.97–1.05)
2 to < 4 years	1.01 (0.98–1.03)	1.00 (0.98–1.02)	1.04 (1.00–1.08)	1.02 (0.98–1.07)
4+ years	1.00 (0.98–1.03)	0.99 (0.96–1.01)	**1.08 (1.03–1.12)**	**1.05 (1.00–1.09)**
**ART regimen**				
AZT/NVP/3TC	**0.97 (0.94–0.99)**	**0.97 (0.95–0.99)**	**0.97 (0.94–0.99)**	0.97 (0.95–0.99)
AZT/EFV/3TC	1.00 (0.97–1.03)	1.00 (0.97–1.03)	0.97 (0.94–1.01)	0.97 (0.93–1.00)
TDF/NVP/3TC	0.98 (0.95–1.01)	0.97 (0.95–1.00)	1.00 (0.97–1.03)	0.99 (0.96–1.02)
PI-based	**1.20 (1.15–1.26)**	**1.19 (1.14–1.24)**	1.02 (0.98–1.05)	1.00 (0.97–1.04)
TLE	0.99 (0.98–1.01)	0.99 (0.98–1.01)	0.98 (0.96–1.00)	1.00 (0.98–1.03)
Other	Ref	–	Ref	–

Sensitivity analyses were performed examining the association of support group attendance reported at the current and previous study visits with viral suppression and ART adherence across all visits except for enrollment, to better characterize the association between longevity of attending support groups and the outcomes of interest. Support group attendance reported at the two consecutive visits was associated with complete ART adherence in unadjusted analyses, but this association was not observed after controlling for potential confounders. No association was observed between support group attendance reported at the two consecutive visits and viral suppression in unadjusted and adjusted analyses. Statistically significant ORs and 95% CI are in bold

*OR* odds ratio, *CI* confidence interval

### Viral suppression

Across all study visits, 645/19,538 (3.3%) viral load test results were missing in PLWH on ART. Viral load < 1000 copies/mL was documented in 91.9% of PLWH on ART with complete data across all study visits; viral failure (≥1000 copies/mL and on ART > 6 months) was observed in 7.6%. Across all study visits, participants who reported complete ART adherence were significantly less likely to have viral failure than those who had missed one or more doses of ART in the past 30 days (6.8% vs. 13.9%, *p* < 0.01).

As compared to PLWH who did not report support group attendance, those who did had similar odds of viral suppression in unadjusted (OR 0.99, 95% CI 0.97–1.01) and adjusted analyses (aOR 0.99, 95% CI 0.97–1.01).

Sensitivity analyses showed no association between support group attendance at the current and previous visit and viral suppression across all visits in unadjusted (OR 0.99, 95% CI 0.97–1.01) and adjusted analyses (aOR 0.99, 95% CI 0.97–1.01) (Table 3).

## Discussion

Participation in support group meetings offers PLWH the opportunity to contribute to their care with a view to improving their treatment outcomes. We assessed the impact of HIV support group participation on ART adherence and viral suppression among adult PLWH on ART in four sub-Saharan African countries and found that support group participation was generally low and was not significantly associated with ART adherence or viral suppression.

Available data on uptake of HIV support groups in SSA are mixed. Consistent with our observation, low uptake of HIV support groups ranging from 5.2–27% has been reported by several previous studies in SSA [15, 21–23]. However, other studies in SSA have reported much higher support group uptake, ranging from 46 to 53% [7, 17, 24]. As seen in our cohort, significant differences in inter-site HIV support group uptake was reported in a multi-site randomized behavioral intervention trial in the U.S. [22] We observed significant within-site differences in support group attendance at nearly all the sites. This differs from observations in a public sector ART program in South Africa where uptake of support groups did not significantly change over 24 months of follow-up among 268 PLWH [15]. Differences in the characteristics of the study population and the definition of HIV support group attendance might have contributed to the observed discrepancy in support group uptake. Given the multi-country spread and the large cohort involved in this study, our findings suggest that low uptake of support groups at various HIV care centers in SSA is a problem that needs to be addressed.

This study showed that HIV support group attendance was neither significantly associated with ART adherence nor viral suppression. In agreement with our observation, a cluster randomized controlled trial in Vietnam that focused on virologic outcome found no association between support group attendance and viral suppression [18]. Participation in an HIV support group has been associated with significantly improved ART adherence and viral suppression in a number of prior studies that either assessed both or one of the outcomes [7, 15–17].

Although we found that more frequent support group attendance measured by a self-report of support group attendance at the current and previous visits was significantly associated with improved ART adherence in unadjusted analysis, this association was not observed after controlling for potential confounders. More frequent support group attendance also did not demonstrate any measured improvement in viral suppression in our cohort. There have been variable observations in the few available literature that investigated the impact of frequency of support group participation on ART adherence and viral suppression. Contrary to our findings, PLWH in Central Kenya who reported more frequent participation in HIV support grpoup meetings were more likely to have better ART adherence and viral suppression [16]. Despite reporting better ART adherence among support group attendees compared to non-attendees, a previous study in Nigeria failed to demonstrate an association between more frequent support group attendance and ART adherence [7].

Various factors may be responsible for the observed differences. High support group uptake was a common observation among studies that demonstrated improved ART adherence and/or viral suppression among support group attendees compared to non-attendees [7, 16, 17], which may partly explain the disparity with our findings. In addition, two of those studies were limited by small sample sizes while the third was a cross-sectional analysis. The well-known lack of standardization in the services provided by HIV support groups may also contribute to the observed disparity. Differences in ART duration and regimen in the various studies may also play a role. The high frequency of self-reported ART adherence in our participants could partly explain the lack of association with support group attendance. Moreover, many of the participants were already engaged in care prior to their study enrollment so it is possible that good treatment outcomes that may be attributable to participation in support groups could have antedated the study. Detectable HIV viral load is associated with infective and non-infective complications [25–27]; hence a higher tendency to achieve viral suppression should be targeted as a treatment outcome goal among HIV support group participants.

Despite the lack of association between support group attendance and ART adherence or viral suppression, the well-known interdependence between ART adherence and viral suppression was corroborated in our study. PLWH who reported complete ART adherence were significantly more likely to have viral suppression. In order to achieve improved HIV treatment outcomes, strategies aimed at promoting ART adherence should continue to be intensified during HIV support group sessions.

Our analyses should be interpreted in the light of their limitations. In the bid to make our findings as representative as possible of the HIV clinic patients at each site, an overwhelming majority of our participants were randomly selected from existing patient list at each clinic stratified by gender, and ART status. However, once a participant enters the cohort, they begin to receive an enhanced level of care and may no longer be representative of the routine care experience which could potentially affect the generalizability of our findings. While we provided a general description of known support group operations at the sites, we were not able to determine the extent to which subtle differences in these operations impacted our outcomes since the data were not systematically collected as part of the study procedures. Although we assessed the impact of support group attendance at the current and previous visit on viral suppression and ART adherence across all visits, this might not have clearly delineated those who consistently attended support groups from those with infrequent attendance. ART adherence was based on self-report, which potentially undermines the internal validity. Although we were able to control for WHO clinical stage, ART duration, regimen and the existence of an ART treatment supporter, this study did not collect information on drug interactions and side effects, so our analyses were unable to control for these additional potential confounders. It is possible that the high frequency of self-reported ART adherence and viral suppression in the cohort on a background of low support group uptake limited our ability to detect any potential effect of support group participation. Considering that several participants were already engaged in care prior to their study enrollment, it is possible that the impact of support group attendance on the primary outcome measures might have been experienced prior to enrollment in this study.

## Conclusion

We found that HIV support group participation was uncommon in our cohort and was not significantly associated with ART adherence or viral suppression. Considering that uptake of support group in the four sub-Saharan African countries assessed was low, our findings possibly highlight how the potential benefits of HIV support groups might be hindered by low uptake in a region with disproportionately high burden of HIV, a critical need for good ART adherence and viral suppression, both of which are components of the ‘95–95-95 targets’. While the need for increased awareness of support groups and their potential benefits among PLWH in SSA cannot be overemphasized, comprehensively exploring the factors responsible for low HIV support group uptake in the region should be pursued.

## Data Availability

The Henry M. Jackson Foundation for the Advancement of Military Medicine (HJF) and the U.S. Department of the Army are committed to safeguarding the privacy of all research participants. The study investigators and ethical review committees have implemented necessary measures to ensure participant anonymity is maintained in all reporting of research data. Distribution of de-identified participant-level data and accompanying research resources will require compliance with all applicable regulatory and ethical processes. Requests for these materials can be made via e-mail to PubRequest@hivresearch.org.

## Abbreviations

AFRICOS: African Cohort Study
ART: Antiretroviral Therapy
CI: Confidence Interval
HIV: Human Immunodeficiency Virus
OR: Odds Ratio
PEPFAR: President’s Emergency Plan for AIDS Relief
PLWH: People Living With HIV
SRV: South Rift Valley
SSA: Sub-Saharan Africa
UNAIDS: Joint United Nations Program on HIV and AIDS
WHO: World Health Organization
